# 

**Published:** 1947

**Authors:** 


					I,
Vol. LXIV. No. 231.
I-
AUTUMN, 1947.
The Bristol
Medico-Chirurgical Journal
PUBLISHED UNDER THE AUSPICES OP
THE BRISTOL MEDICO-CHIRURGICAL SOCIETY.
Editor: J. A. NIXON, C.M.G., M.D., F.R.C.P.
WITH WHOM ABE ASSOCIATED
A. RENDLE SHORT, M.D., B.S., B.Sc., F.R.C.S.
W. MELVILLE CAPPER, M.B., B.S., F.R.C.S.
G. E. F. SUTTON, M.C., M.D., B.S., M.R.C.P.
E. WATSON-WILLIAMS, M.C., Ch.M., F.R.C.S., Editorial Secretary.
BRISTOL:
J. W. ARROWSMITH LTD., QUAY STREET & SMALL STREET
Price Four Shillings net.

				

